# Supplementary material for: Gentirigeoside B from Gentiana rigescens Franch Prolongs Yeast Lifespan via Inhibition of TORC1/Sch9/Rim15/Msn Signaling Pathway and Modification of Oxidative Stress and Autophagy
Source: Antioxidants (Basel). 2022 Nov 30;11(12):2373. doi: 10.3390/antiox11122373 (PMC9774393; doi:10.3390/antiox11122373)
Supplement: Supplementary file 1 [file antioxidants-11-02373-s001.zip › antioxidants-2040797-supplementary.pdf]

Supplementary Information

Gentirigeoside B from *G. rigescens* Franch Prolongs Yeast Lifespan via Inhibition of TORC1/Sch9/Rim15/Msn Signaling Pathway and Modification of Anti-Oxidative Stress and Autophagy

Lan Xiang[1*], Dejene Disasa [1&], Yanan Liu[1&], Rui Fujii[2], Mengya Yang[1], Enchan Wu[1], Akira Matsuura[2] and Jianhua Qi[1*]

1. College of Pharmaceutical Science, Zhejiang University, 866 Yu Hang Road, Hangzhou, China, 11719053@zju.edu.cn (D.D.); liuyanan1231@zju.edu.cn (Y. L.); 11819026@zju.edu.cn (M.Y.); 22019019@zju.edu.cn (E.W.)

2. Department of Biology, Graduate School of Science, Chiba University, Chiba 263-8522, Japan, afga1829@chiba-u.jp (R.F.); amatsuur@faculty.chiba-u.jp (A.M.)

[&] Represents equal contribution

*Correspondence should be addressed to Lan Xiang (lxiang@zju.edu.cn); Jianhua Qi (qijianhua@zju.edu.cn); Tel and Fax: +86-571-8820-8627

Supplementary Table S1. List of primers sequence used for RT-PCR study.

Genes	Species	Sequences	Gene ID
SOD1	Yeast	sense: 5′-CAC CAT TTT CGT CCG TCT TT-3′<br>antisense 5′-TGG TTG TGT CTC TGC TGG TC-3′	853568
SOD2	Yeast	sense: 5′-CTC CGG TCAAAT CAA CGA AT-3′<br>anti-sense: 5′-CCT TGG CCAGAA GAT CTG AG-3′	856399
GPx	Yeast	anti-sense: 5′-CCT TGG CCAGAA GAT CTG AG-3′<br>sense: 5′-CGC TCC GTC AAG TAA ACA TAG G-3′	S000001509
CAT	Yeast	anti-sense:5′-GGC CGC TGT TAT TGT TTT GAA C-3′<br>sense: 5′-TGA CAA ACT CCA CTG GTA ATC C-3′	S000002664
TUB1	Yeast	anti-sense: 5′-TCC CTG TTG AAA TGA GCC AA-3′<br>sense: 5′-CCA AGG GCT ATT TAC GTG GA-3′	854889
TORC1	Yeast	sense: 5‘-TTG GTA CAA GGC ATG GCA TA-3'<br>anti-sense: 5'-TAC CGT CAA TCC GCA CAT TA-3'	S000218103
SIR2	Yeast	sense: 5'-CGT TCC CCA AGT CCT GAT TA-3'<br>anti-sense: 5'-CCA CAT TTT TGG GCT ACC AT-3′	851520
RPS26A	Yeast	sense: 5'-TCA GAA ACA TTG TTG AAG CCG C-3'<br>anti-sense: 5′-ACA ATT CTG GCG TGA ATA GCA C-3'	852686
RPL9A	Yeast	sense: 5'-ATG GTG CCA AAT TCA TTG AAG TC-3'<br>anti-sense 5'-AGTTACCTGACAAGACAATTTCG-3'	852730
SCH9	Yeast	sense:5'-GCC ATT GAT GAT GGA AGA GAA CTAC-3'<br>anti-sense:5'- TCATATTTCGAATCTTCCACTGAC-3'	856612
RIM15	Yeast	sense: 5'-GGA GCT GGA ACT GGA CGG CAAG -3'<br>anti-sense: 5'-AGC ATG TCT GTG GCC TTT TGAA-3'	850511
MSN2	Yeast	sense: 5'-AGA ACG ATA TGC TGC CGA ATTC-3'<br>anti-sense: 5'-CGCCACTTTCGCAATAACG-3'	855053
MSN4	Yeast	sense: 5'-GGATTGATGGACCCGGTATTG-3'<br>anti-sense: 5'-CCAAAGGTATATTCCGGCGAA-3'	853803

Supplementary Table S2. Yeast strains were used in the present study.

Strains	Genome-type	Source
K6001	MAta, Ade2-1, trp1-1, can1-100, leu2-3, 112, his-3-11, 15, GAL, psi+, ho::HO::CDC6 (at HO), cdc6::hisG, URA3 GAL-ubiR-CDC6 (URA3)	Gifted by Prof. Michael Breitenbach
Δ*uth1,* Δ*skn7,* Δ*cat,* Δ*gpx,* Δ*sod1,* Δ*sod2* with K6001 background	Replacement of UTH1, SKN7, SOD1 and SOD2 with Kanamycin gene.	Constructed by professor Akira Matsuura
BY4741	Mata, his3Δ1, Leu2Δ0, met15Δ0, ura3Δ0	Constructed by professor Akira Matsuura
MSN2-GFP yeast with BY4741 background	Mata, his3Δ1, Leu2Δ0, met15Δ0, ura3Δ0；MSN2-GFP::KanMX	Constructed by professor Akira Matsuura
sfGFP-SCH9 yeast with BY4741 background	Mata, his3Δ1, Leu2Δ0, met15Δ0, ura3Δ0；sfGFP-SCH9-5HA::LEU2	Constructed by professor Akira Matsuura
RIM15-GFP yeast with BY4741 background	Mata, his3Δ1, Leu2Δ0, met15Δ0, ura3Δ0，RIM15-GFP::His3MX	Constructed by professor Akira Matsuura
YOM36	Prototrophic derivative of BY4742 (MATα, his3Δ1, leu2Δ0, lys2Δ0, ura3Δ0)	Gifted by professor Akira Matsuura
YOM38 containing plasmid pR316-GFP-ATG8	Prototrophic derivative of BY4742 (MATα, his3Δ1, leu2Δ0, lys2Δ0, ura3Δ0) containing plasmid pR316-GFP-ATG8	Constructed by professor Akira Matsuura

Supplementary Figures

^{13}C NMR for GTS B molecule (Pyridin-d_5)

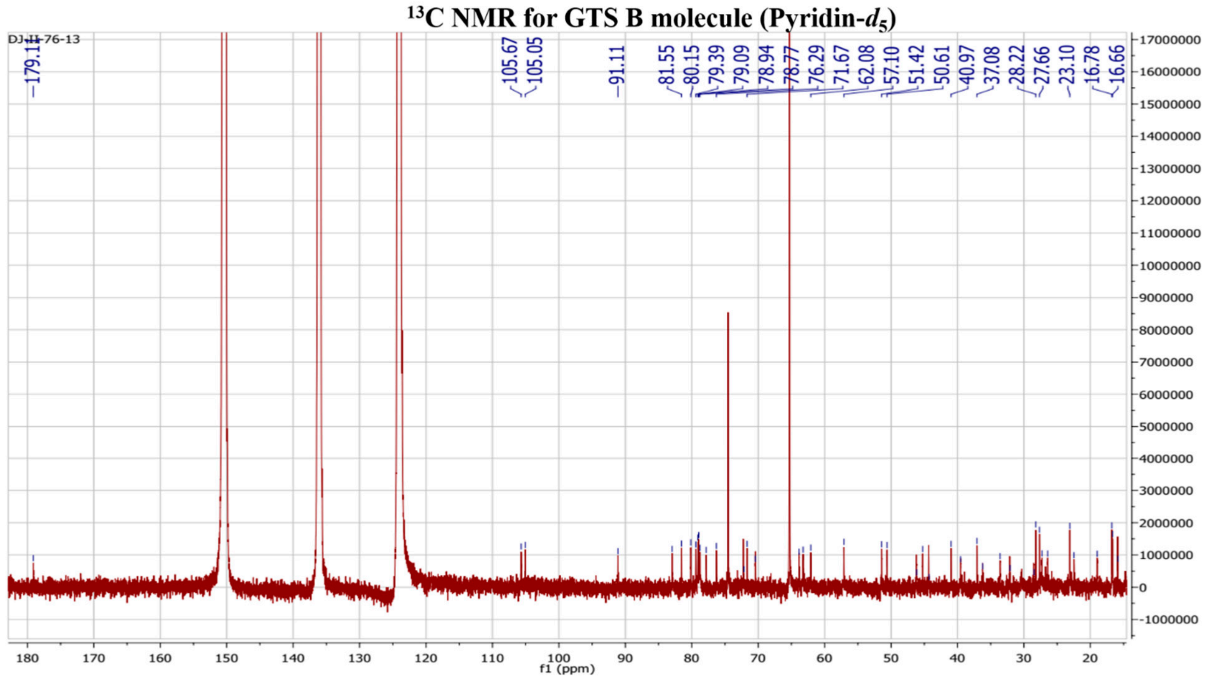

Supplementary Figure S1. The ^{13}C NMR spectrum of Gentirigeoside B from *G. rigescens* Franch L.

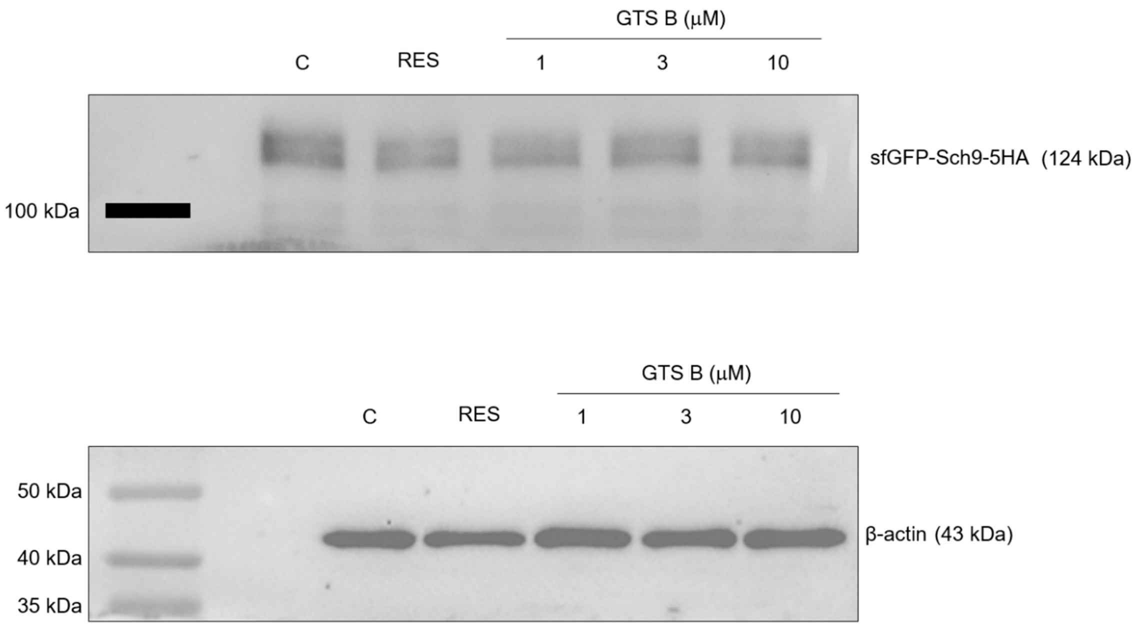

Supplementary Figure S2. The original results of western blotting analysis for Sch9 in Figure 3(b).
